# Aetiology of femoral hernias revisited: bilateral femoral hernia in a young male (two cases)

**DOI:** 10.1308/003588413X13511609955733

**Published:** 2013-01

**Authors:** RT Kouchupapy, G Ranganathan, S Dias, D Shanahan

**Affiliations:** ^1^Prince Philip Hospital, Llanelli,UK; ^2^West Wales General Hospital, CarmarthenUK

**Keywords:** Bilateral femoral hernia, Femoral hernia in young male, Femoral hernia aetiology

## Abstract

Bilateral femoral hernias are less common in men than in women and rare in young adults. Only one case of a bilateral femoral hernia in a young man has been reported in the literature before. Three main theories have been postulated for femoral hernias. The theory that they are an acquired disease is the most accepted due to the common occurrence of such hernias in multiparous women but the theory lacks enough evidence. We report two cases in young men. Anatomical variations in the femoral canal could be the primary aetiological factor in these patients. A unilateral femoral hernia in young men with acquired aetiological factors requires a clinical examination of the opposite side.

Femoral hernias are the second most common groin hernias in adults, with an incidence of 2–4%.^1^ They are more common in women than in men.^2^ Bilateral femoral hernias in young men are rare.^3^ Femoral hernias often present as an incarcerated or strangulated hernia.^4^ There is an increased morbidity and mortality from delayed presentation and treatment. Awareness is therefore crucial for the diagnosis of a condition with such a low incidence in young males.

## Case 1

A 24-year-old man working as a hospital porter was seen in the accident and emergency department with a 1-day history of right groin swelling associated with discomfort and pain. He was afebrile and his body mass index (BMI) was 24kg/m^2^. An examination of his groin showed an irreducible tender globular swelling that was located below the inguinal ligament. A clinical diagnosis of an incarcerated femoral hernia was made. An examination of the left groin was normal, as were laboratory investigations. After taking informed consent, a femoral hernia repair was performed using the low approach (Lockwood’s operation). The sac contained a preperitoneal pad of fat. The sac was transfixed and reduced. A Vypro® mesh plug was used for the repair and the skin closed with subcuticular Monocryl® sutures.

The patient was reviewed three months later with a two-day history of left groin swelling with discomfort and pain. An examination showed a left incarcerated femoral hernia but no signs of strangulation. A low approach repair was performed with a Vypro® mesh plug and Prolene® sutures. His postoperative recovery was uneventful and he was discharged the following day.

## Case 2

A 40-year-old male with a BMI of 21kg/m^2^ was seen in the accident and emergency department for a painful right groin that had been worsening in intensity over a 2-week period. He complained of having had dysuria and increased urinary frequency for over a year and was treated for prostatitis after investigations with a course of antibiotics and an α-blocker. The patient was known to have a longstanding history of back pain owing to lumbar spondylosis and he is a former smoker.

**Figure 1:**
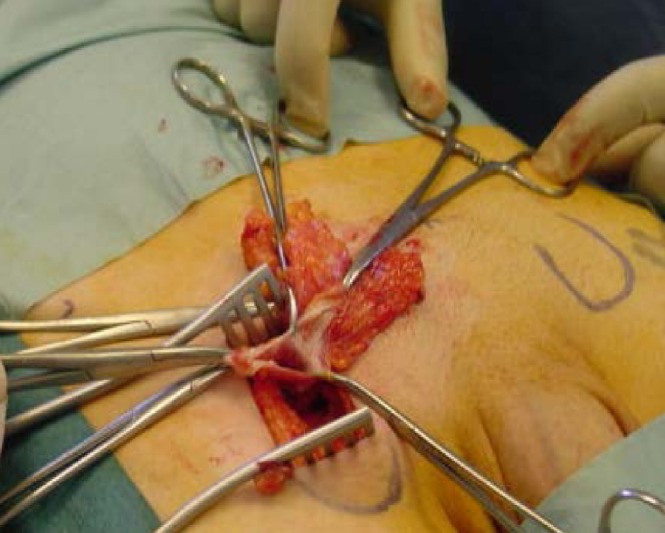
Right femoral hernia sac

**Figure 2:**
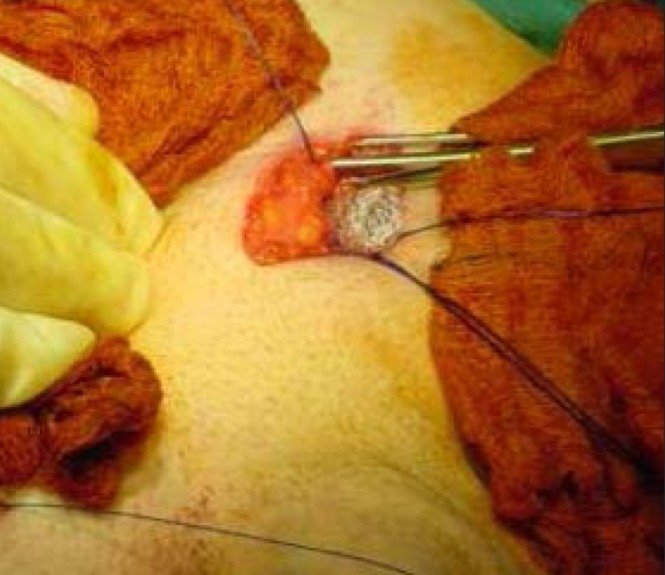
Plug mesh repair of left femoral hernia

An examination of the groin showed a reducible bilateral femoral hernia. Informed consent was obtained from him after a urology opinion. A bilateral femoral hernia repair was carried out using a low inguinal (Lockwood) approach. Both these hernias occurred through the femoral canal and no obvious anatomical abnormality was noted. The sac contained a preperitoneal pad of fat on both sides (Fig 1). A Prolene® mesh plug was placed and fixed with 2/0 Prolene® sutures to the inguinal and pectineal ligament (Fig 2). The skin was closed with 2/0 subcuticular Prolene® sutures. His postoperative recovery was uneventful and he was discharged the following day.

## Discussion

Femoral hernias are a protrusion of the peritoneal sac covered with an extraperitoneal pad of fat through the femoral canal. They account for 2–4% of all groin hernias in adults.^1^ The male-to-female ratio is 1:4 and incidence increases with age.^2^ The majority of hernias occur in women aged over 50 years.^4^ Femoral hernias account for 2% of all hernias in men but they constitute around 24% of all hernias in women.^2^ The right side is affected twice as often as the left and 20% of cases are bilateral. A bilateral femoral hernia in a young man has only been reported once before.^3^

The fundamental aetiology of femoral hernias is an enlarged femoral ring.^5^ The lacuna vasorum increases in size in both sexes from birth to old age. This may account for the increased incidence in elderly patients.^4^ The lacuna musculorum decreases in size with age, possibly explaining the comparatively increased incidence in elderly men. Theories on the cause of femoral hernias include the existence of: a congenital preformed sac, an acquired aetiology and anatomical variations of the femoral canal together with acquired aetiological factors.

Keith discredited the preformed sac theory in 1923.^6^ He stated that the femoral ring is a kind of safety valve that allows the expansion of the femoral vein in the upright posture. He also emphasised that any weak areas in the abdominal wall will develop hernias owing to the constant repetition of increased intra-abdominal pressure.

The theory that femoral hernias are an acquired disease has been accepted owing to the predominance of these hernias in multiparous women. It is thought to be due to increased intra-abdominal pressure and the stretching of aponeurotic tissue. Obesity has been considered as one of the aetiological factors for groin hernias but evidence from 2008 showed it might actually be protective of such hernias.^7^ However, according to Tasche, the available space for the development of a femoral hernia is smaller in women than in men.^8^ Both of our patients had increased abdominal pressure for a long period of time and a low BMI.

Anatomical study showed a large normal variation in the breadth of the attachment of the posterior inguinal wall, which may affect the diameter of the femoral ring inversely.^5^ A non-existent iliopubic tract or pectineal ligament are notable anatomical variations. The anatomical variation of the narrow posterior inguinal wall attachment on to the pectineal ligament, resulting in an enlarged femoral ring, may be the primary aetiological factor, according to Tasche.^8^ Anatomical variations in individuals can precipitate the development of femoral hernias in the presence of acquired aetiological factors.^5^

The ‘congenital’ theory has been discredited owing to a lack of evidence and the fact that femoral hernias are not common in young adults. There is also no strong evidence for the ‘acquired disease’ theory. An acquired aetiology is more likely to be a precipitating rather than a primary factor as there is not enough evidence. A preformed sac with possible acquired factors has been the accepted aetiology for indirect inguinal hernias. Anatomical variations of the femoral canal together with acquired aetiological factors might be a possible explanation for femoral hernias. Both our patients had aetiological factors for the increased intra-abdominal pressure. One explanation for these cases is that there may have been prior anatomical variations that could have precipitated a hernia with acquired aetiological factors.^8^

## Conclusions

A unilateral femoral hernia in young, thin built men should raise suspicions about the opposite side. Bilateral femoral hernias need to be repaired at the same time to avoid morbidity.
